# Nanoplatforms for Sentinel and Tumor‐Draining Lymph Node Mapping: From Visualization to Pathway‐Informed Staging

**DOI:** 10.1002/adhm.71423

**Published:** 2026-07-11

**Authors:** Xu Chen, Nina Li, Meiyan Zou, Zihao Zhou, Rongwei Xu, Weiyao Feng, Xinyuan Zhao, Li Cui

**Affiliations:** ^1^ Stomatological Hospital, School of Stomatology Southern Medical University Guangzhou Guangdong China

**Keywords:** lymphatic mapping, multimodal imaging, nanoparticle tracers, sentinel lymph nodes, tumor‐draining lymph nodes

## Abstract

Accurate identification of sentinel lymph nodes (SLNs) and tumor‐draining lymph nodes (TDLNs) is central to oncologic staging, treatment stratification, and surgical decision‐making. However, conventional radiotracer‐ and dye‐based approaches remain limited by suboptimal signal specificity, inflexible workflows, and reduced reliability in anatomically complex or therapy‐altered lymphatic networks. Nanoparticle‐based lymphatic tracers address these limitations by enabling controllable lymphatic transport, stable intranodal retention, and multimodal signal generation, thereby expanding the precision and applicability of SLN/TDLN detection. This review analyzes recent advances in carbon‐based, magnetic, metal/metal‐oxide, polymeric/organic, and bio‐derived or biomimetic nanoparticles for lymph node mapping, with emphasis on how material composition, surface engineering, and physical contrast mechanisms translate into clinically actionable imaging performance. Shared design principles underlying three key objectives are further synthesized: sensitive detection of early and occult nodes, reliable discrimination of metastatic involvement with improved staging accuracy, and robustness across delayed surgery, neoadjuvant therapy, and multi‐step clinical workflows. Collectively, these advances position lymphotropic nanoparticles as a mechanistically grounded and clinically promising platform for extending both the accuracy and operational boundaries of SLN/TDLN assessment.

## Graphical abstract

Lymphotropic nanoplatforms integrate multimodal imaging with therapeutic guidance to map sentinel and tumor‐draining lymph nodes, revealing functional drainage, metastatic involvement, and nodal accessibility, while providing mechanistic insight to support precision surgical and therapeutic interventions.

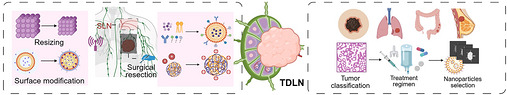

## 1 Introduction

Lymph node metastasis represents a pivotal determinant of disease progression and prognosis across a wide spectrum of solid malignancies. Within this framework, the sentinel lymph nodes (SLNs) and tumor‐draining lymph nodes (TDLNs) occupy a central role in pathological staging, surgical decision‐making and the rational tailoring of locoregional therapy [1, 2, 3]. Accurate identification and assessment of these nodes directly inform the extent of lymphadenectomy, the use of neoadjuvant or adjuvant treatment, and strategies aimed at balancing oncological control against surgical morbidity [4, 5]. As a result, reliable SLN/TDLN detection remains a cornerstone of contemporary oncologic management.

**FIGURE 1:**
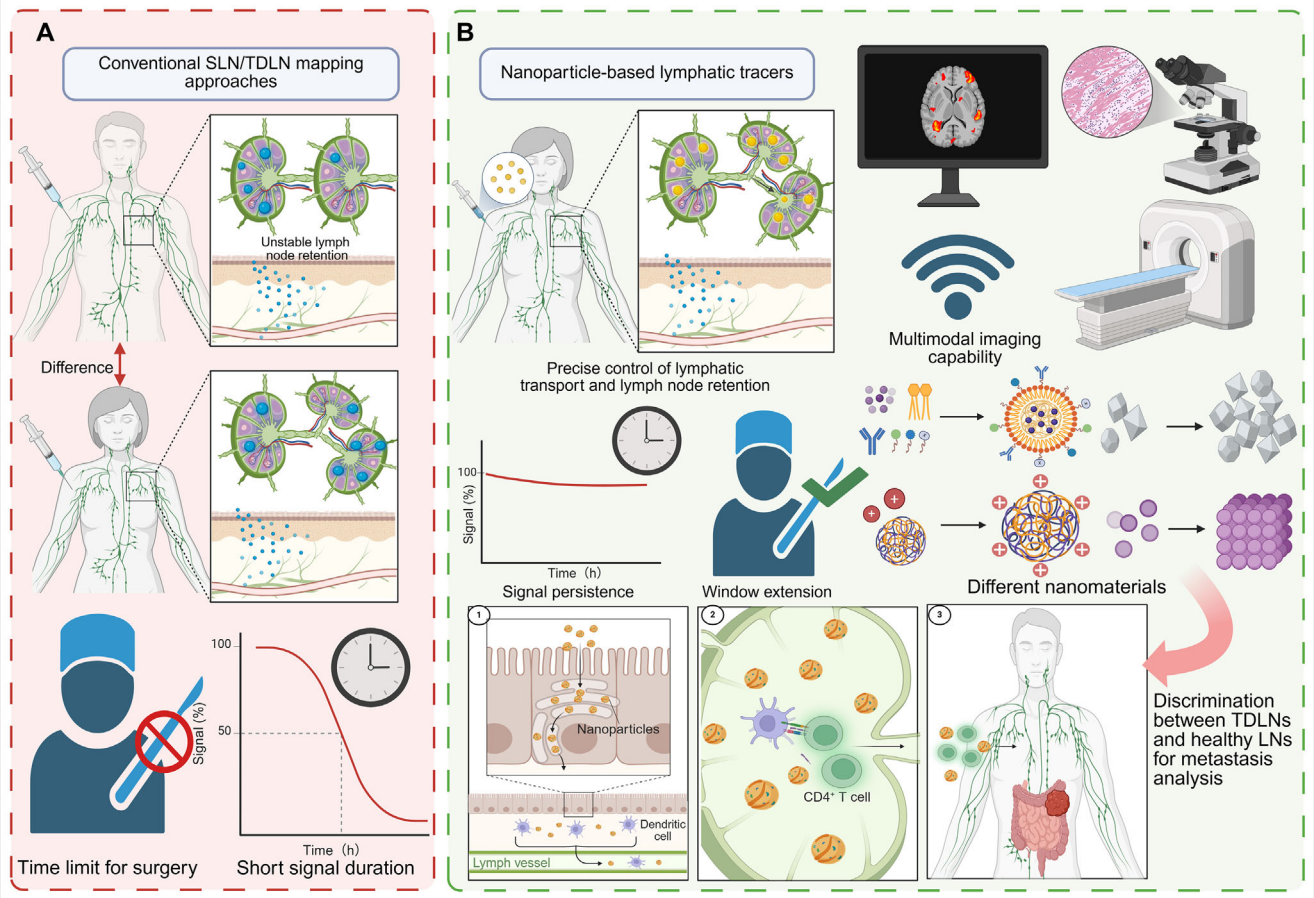
Limitations of traditional lymph node imaging and advantages of nanoparticle‐based tracers. (A) In some clinical settings, signal persistence and variable nodal retention may constrain the surgical time window, while complex anatomy and interpatient lymphatic variability can affect detection consistency. (B) Featuring precisely controlled lymphatic transport, stable nodal retention and multimodal imaging; engineered to overcome limitations of conventional methods for accurate discrimination between tumor‐draining and healthy lymph nodes in metastasis analysis.

Despite widespread clinical adoption, conventional SLN/TDLN mapping approaches based on radiocolloids, vital dyes or fluorescent tracers remain constrained by tracer‐dependent kinetics, depth sensitivity and workflow requirements. In radiocolloid lymphoscintigraphy, dynamic imaging partly addresses these limitations by tracking lymphatic collectors and helping distinguish true first‐echelon nodes from downstream second‐tier uptake, while small particle radiolabelled tracers such as ^99m^Tc‐labelled antimony sulphide colloid provide favorable lymphatic‐channel visualization and limited onward migration after early nodal uptake [6, 7]. However, these advantages depend on optimized tracer selection and imaging protocols and are not shared equally by vital dyes or fluorescent tracers, which can be limited by transient signals, tissue‐depth constraints, equipment dependence or single‐modality readouts [8]. Such technical limitations can further complicate nodal interpretation in anatomically complex basins, in patients with variable lymphatic drainage, or after lymphatic remodeling induced by neoadjuvant therapy [9] (Figure 1A).

Nanoparticle‐based lymphatic tracers have emerged as a powerful alternative paradigm for SLN/TDLN detection by offering precise control over lymphatic transport, nodal sequestration and signal output [10, 11]. Through rational engineering of particle size, surface chemistry, ligand functionalization and material composition, nanoplatforms enable predictable drainage along afferent lymphatics, stable intranodal retention and multimodal imaging capability [12, 13, 14]. Importantly, the physical and chemical properties conferred by distinct nanomaterial classes—ranging from magnetic and optical to acoustic and radiological—extend nodal mapping beyond mere localization, facilitating discrimination of true tumor‐draining nodes and interrogation of metastatic involvement with improved spatial and temporal resolution [15, 16] (Figure 1B).

In this review, we synthesize recent advances in nanoparticle‐enabled SLN/TDLN detection, encompassing carbon‐based nanotracers, magnetic inorganic nanoparticles, metal and metal‐oxide nanoplatforms, polymeric and organic systems, and bio‐derived or biomimetic nanoparticles. By examining representative studies across early and occult node detection, metastasis‐sensitive nodal discrimination and adaptability to complex clinical workflows, we distil shared design principles and mechanistic insights underpinning effective lymphatic navigation. Collectively, these developments highlight how nanotechnology is reshaping SLN/TDLN mapping from a procedural adjunct into a flexible, information‐rich component of precision oncologic care.

## 2 Carbon‐Based Nanoparticles as Lymphotropic Probes for Resilient SLN/TDLN Detection and Staging

**FIGURE 2:**
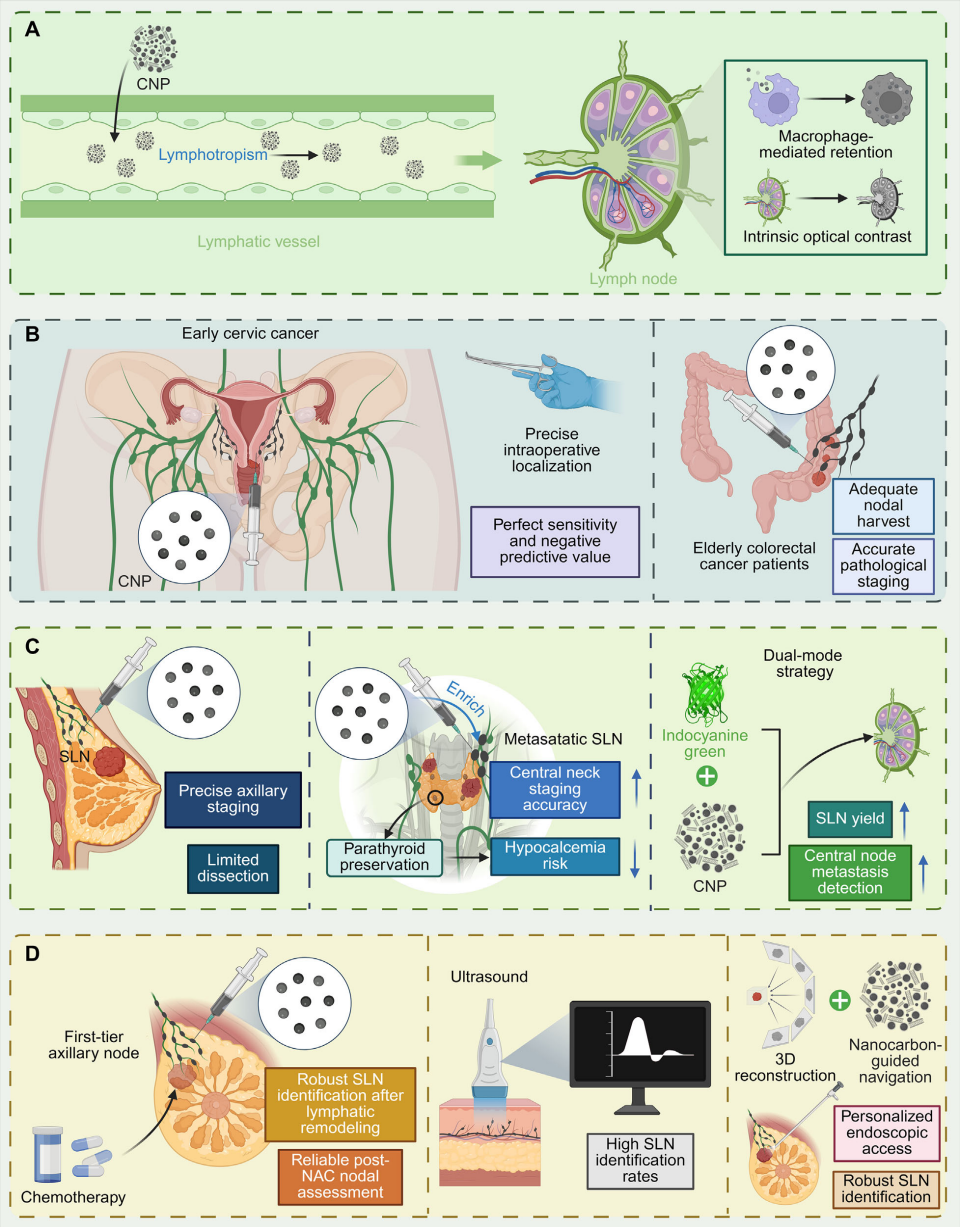
Carbon‐based nanoparticles as resilient lymphotropic probes for sentinel and tumor‐draining lymph node mapping. (A) Fundamental lymphatic behavior of carbon‐based nanoparticles (CNPs). Owing to their optimized nanoscale size and surface properties, CNPs rapidly enter lymphatic vessels after local injection and preferentially accumulate within draining lymph nodes through macrophage‐mediated retention, providing stable intranodal deposition and intrinsic optical contrast for node visualization. (B) Clinical application of CNPs for sensitive SLN detection and accurate pathological staging across solid tumors. In early cervical cancer, cervical stromal injection of CNPs enables rapid lymphatic transit and intense black staining of SLNs, allowing precise intraoperative localization and comprehensive delineation of pelvic drainage pathways with perfect sensitivity and negative predictive value. In elderly colorectal cancer patients, CNP tracing selectively highlights early‐draining abdominal SLNs, including sub–2‐mm and micrometastatic nodes frequently missed by conventional dissection, thereby reducing inadequate nodal harvest and improving staging accuracy. (C) Precision nodal staging and dual‐mode strategies in anatomically complex settings. In early breast cancer, CNP suspensions achieve near‐universal intraoperative SLN visualization with high specificity, enhancing pathological contrast within metastatic nodes while limiting unnecessary axillary dissection. In papillary thyroid microcarcinoma, combined use of indocyanine green and CNPs integrates real‐time fluorescence guidance with durable intranodal carbon deposition, increasing SLN yield and improving detection of metastatic central compartment nodes compared with single‐modality approaches. (D) Robust performance of CNP‐based mapping under perturbed or advanced clinical workflows. Following neoadjuvant chemotherapy, CNPs reliably accumulate within first‐tier axillary nodes despite treatment‐induced lymphatic remodeling, enabling dependable post‐NAC nodal assessment. Ultrasound‐assisted CNP mapping further sustains high SLN identification rates through real‐time visualization of carbon‐laden nodes when optical or radioactive tracers are impractical. Integration of nanocarbon‐guided navigation with three‐dimensional reconstruction supports patient‐specific endoscopic access while maintaining robust SLN detection, underscoring the adaptability of carbon‐based probes across minimally invasive and workflow‐complex surgical contexts.

Carbon‐based nanoparticles have emerged as a mature and versatile class of lymphotropic probes for SLN or TDLN detection, driven by their favorable size range, intrinsic optical contrast, and stable macrophage‐mediated nodal retention [17, 18] (Figure 2A). Across a broad spectrum of solid tumors, their performance is reflected in three interrelated aspects: the capacity to uncover early or otherwise occult lymphatic targets [19], the precision with which metastatic involvement and nodal status are resolved [20, 21], and the resilience of nodal mapping in anatomically or therapeutically perturbed clinical contexts [22]. These aspects span the continuum from initial lymphatic access and sensitive node discovery, through accurate pathological staging, to reliable application after neoadjuvant therapy or in minimally invasive workflows.

### 2.1 Early and Occult Sentinel Lymph Node Detection Enabled by Lymphotropic Carbon Nanotracers

Early and occult nodal detection constitutes a core advantage of carbon‐based nanotracers, as their intrinsic lymphotropism and high‐contrast pigmentation enable visualization of SLNs at anatomical scales or disease stages that frequently escape conventional techniques [23, 24]. By preferentially accessing first‐echelon lymphatics and accumulating within early‐draining or microscale nodes, carbon nanoparticles convert subtle lymphatic flow patterns into robust intraoperative readouts, supporting sensitive SLN identification across diverse tumor settings [25]. For example, carbon nanoparticles injected into the cervical stroma rapidly traverse regional lymphatics and generate intense black staining that enables precise intraoperative localization of sentinel nodes in early cervical cancer, accurately delineating obturator, internal, and external iliac drainage pathways with perfect sensitivity and negative predictive value [26]. Similarly, in elderly colorectal cancer patients, carbon nanoparticle tracing selectively highlights early‐draining abdominal SLNs, including micrometastatic nodes often missed by standard dissection, thereby increasing recovery of sub–2‐mm nodes, reducing inadequate nodal harvest, and improving pathological staging accuracy [27] (Figure 2B). In addition, nanocarbon tracers used for axillary mapping in early breast cancer traffic selectively through first‐draining lymphatics to produce dense pigmentation, achieving near‐complete detection rates and maintaining strong concordance with axillary metastasis even when only limited numbers of SLNs are retrieved [28]. Furthermore, intraoperative carbon nanoparticle application in elderly colorectal cancer markedly increases both total and small‐node yield by preferentially staining micro–lymph nodes that dominate early lymphatic drainage, thereby strengthening SLN/TDLN assessment and supporting tailored lymphadenectomy without compromising detection fidelity [29].

### 2.2 Carbon Nanoparticle–Enabled Concordance between Sentinel‐Node Status and Metastatic Burden

Accurate identification of metastatic involvement and faithful nodal staging constitute a second major strength of carbon‐based nanotracers. Owing to their stable intranodal retention and high‐contrast pigmentation, carbon nanoparticles enhance the visual identification and retrieval of sentinel or tumor‐draining lymph nodes, thereby strengthening pathology‐based concordance between sampled nodes and regional nodal status across gynecologic, breast and endocrine malignancies, including minimally invasive and image‐guided settings. Their staging value therefore lies in converting lymphatic drainage into reliably retrievable nodes for histopathological assessment, rather than relying on carbon staining alone as a surrogate for metastatic involvement [30, 31]. For example, in early breast cancer, carbon nanoparticle suspensions achieved near‐universal intraoperative SLN visualization with a 99% detection rate and perfect specificity, supporting precise axillary staging through pathological assessment of carbon‐stained SLNs rather than metastasis‐selective carbon accumulation [32]. Similarly, during laparoscopic staging of endometrial cancer, carbon nanoparticles provided stable, high‐contrast delineation of uterine lymphatic drainage, with cervical injection yielding superior bilateral SLN detection and accurate identification of metastatic nodes. By contrast, fundal injection—although traceable by carbon nanoparticles—revealed an additional infundibulopelvic drainage pathway but was associated with reduced accuracy and higher false‐negative rates, thereby defining anatomical constraints on reliable SLN/TDLN assessment rather than limitations of the nanotracer itself [33]. In addition, carbon nanoparticle–guided SLN mapping in early cervical cancer delineated both typical and atypical pelvic drainage routes, capturing all metastatic spread with a 0% false‐negative rate under laparoscopic conditions [34]. Furthermore, in endometrial carcinoma, rapid lymphatic traversal and preferential labeling of small (≤0.5 cm) nodes enabled carbon‐laden SLNs to achieve perfect sensitivity, specificity, and overall staging accuracy [35].

Beyond gynecologic malignancies, carbon nanotracers further refine nodal staging in anatomically constrained or surgically sensitive regions. In parallel, in a clinical study of breast‐approach endoscopic thyroidectomy, intrathyroidal nanocarbon injection stained thyroid lymphatic vessels and level VI lymph nodes, enabling visual mapping of thyroid‐draining central compartments and retrieval of more central nodes, including metastatic nodes. Because parathyroid glands remained unstained, nanocarbon also provided useful negative contrast for intraoperative gland recognition and preservation [36]. Moreover, in papillary thyroid microcarcinoma, a dual‐mode strategy combining indocyanine green with carbon nanoparticles yielded complementary lymphatic readouts, in which real‐time fluorescence guidance was coupled with stable intranodal carbon deposition, increasing SLN yield and improving detection of metastatic central nodes compared with carbon nanoparticles alone [37] (Figure 2C).

### 2.3 Carbon Nanoparticle–Mediated Robustness Across Therapy‐Altered and Workflow‐Diverse Settings

Carbon‐based nanotracers exhibit strong robustness for SLN/TDLN detection by maintaining stable lymphatic trafficking, durable intranodal retention, and high‐contrast visualization across heterogeneous anatomical and treatment‐altered conditions. For example, carbon nanoparticles exploit size‐controlled lymphatic drainage and macrophage‐mediated sequestration to achieve high‐contrast SLN visualization at optimized doses and time points, while inducing no detectable structural or molecular toxicity, thereby supporting safe and durable nodal detection [38]. In addition, logistic analyses demonstrate that nano‐carbon–based SLN identification retains high accuracy despite interpatient variability in body mass index, age, and lymphatic accessibility, indicating that detection performance is governed by predictable transport and retention mechanisms rather than fragile procedural parameters [39]. Similarly, dual‐mode tracing that combines carbon nanoparticles with indocyanine green has been explored in a clinical cohort of endometrial cancer, with the combined approach showing the highest overall SLN detection rate and significantly higher bilateral detection than either tracer alone [40]. This complementary visible–fluorescence strategy supports feasible intraoperative nodal localization, while stronger evidence for carbon nanoparticles as visible SLN tracers comes from a subsequent randomized controlled trial showing detection and diagnostic performance comparable to indocyanine green when near‐infrared imaging is unavailable [41].

Beyond baseline robustness, carbon nanotracers remain effective in clinical settings where lymphatic architecture or workflow is altered by prior therapy or intraoperative constraints. For example, following neoadjuvant chemotherapy, carbon nanoparticle suspension enabled feasible SLN identification in breast cancer patients, with acceptable detection, accuracy and false‐negative rates, supporting its use for post‐NAC axillary assessment [42]. In parallel, a phase III randomized trial in early breast cancer showed that ultrasound‐assisted carbon nanoparticle suspension mapping was non‐inferior to dual tracing with carbon nanoparticles plus indocyanine green for SLN identification. In this approach, ultrasound localized target axillary nodes before incision and helped guide dissection of black‐stained nodes, providing an image‐assisted workflow for visible carbon‐based mapping [43]. Furthermore, evidence from a systematic review and meta‐analysis comparing nanocarbon with methylene blue in breast cancer SLNB showed higher pooled diagnostic performance for nanocarbon, supporting its role as an effective dye‐based tracer rather than evidence for longitudinal TDLN marking after nodal downstaging [44]. In parallel, in a rat model, carbon nanoparticle–enhanced photoacoustic/ultrasound imaging enabled non‐radioactive, depth‐resolved axillary SLN localization and guided fine‐needle aspiration simulation, supporting preclinical feasibility for minimally invasive SLN biopsy guidance [45]. Likewise, integration of digital three‐dimensional reconstruction with nanocarbon‐guided navigation in early breast cancer enabled patient‐specific endoscopic access while maintaining robust SLN identification, achieving 100% detection with low false‐negative rates even under workflow‐complex surgical conditions [46] (Figure 2D). Together, these studies define carbon nanoparticles as clinically accessible lymphotropic tracers whose main utility lies in visual node identification, nodal retrieval and pathology‐based staging. Rather than functioning as molecular probes that directly report metastatic status, carbon nanoparticles convert lymphatic drainage into visible and surgically retrievable nodes, thereby supporting SLN/TDLN assessment across breast, gynecologic, colorectal and thyroid cancer settings. This clinically grounded role distinguishes carbon staining from many newer nanoplatforms whose advantages remain primarily demonstrated through experimental imaging or mechanism‐oriented studies.

## 3 Magnetic Inorganic Nanoparticles Enabling Pathway‐Based, High‐Fidelity and Temporally Flexible SLN/TDLN Mapping

Magnetic inorganic nanoparticles enable SLN/TDLN mapping through tightly coupled control of lymphatic transport, intranodal retention, and magnetic signal generation [13, 47]. The superparamagnetic core governs pathway‐based drainage into first‐echelon nodes and supports high‐fidelity, background‐resistant magnetic readouts that are independent of optical or anatomical variability. In addition, prolonged intranodal persistence of iron‐oxide nanoparticles decouples lymphatic marking from surgical timing, providing a mechanistic basis for flexible integration into delayed and multi‐step clinical workflows [48].

### 3.1 Topology‐Resolved Magnetic Nanotracing for Patient‐Specific SLN/TDLN Reconstruction

Magnetic inorganic nanoparticles display a distinctive capacity to reconstruct patient‐specific lymphatic drainage and to redefine SLN/TDLN topology beyond fixed anatomical templates. By coupling lymphotropic superparamagnetic iron oxide nanoparticles (SPIONs) with preoperative MRI lymphography and/or intraoperative magnetometer navigation, these systems resolve individualized first‐echelon pathways that frequently extend beyond standard dissection fields, thereby increasing the likelihood of sampling true SLNs/TDLNs. This topology‐resolving capability is particularly valuable in high‐risk or anatomically complex basins, where accurate delineation of patient‐specific drainage underpins staging precision while constraining unnecessary lymphadenectomy. For example, in a high‐risk prostate cancer cohort, intraprostatic SPION administration enabled lymphatic trafficking along tumor‐draining routes and magnetometer‐guided SLN identification, revealing metastatic nodes, including nodes located outside the conventional extended pelvic lymphadenectomy template. With reported sensitivity and specificity approaching 100%, this magnetic SLN approach provided additional diagnostic value for individualized nodal staging [49]. Similarly, workflows integrating SPIONs with preoperative MRI and intraoperative magnetometer guidance delineated patient‐specific pelvic drainage patterns with high sensitivity, enabling targeted removal of metastatic nodes while avoiding radioisotope‐associated logistical constraints [50]. In addition, SPION‐enhanced MRI–guided pelvic SLN mapping repeatedly demonstrated drainage pathways extending beyond ePLND boundaries, refining the operational definition of first‐echelon nodes based on true lymphatic topology rather than fixed anatomy [51]. In parallel, high‐resolution MR lymphography following intraprostatic SPION injection reconstructed unexpectedly extensive prostate‐draining networks—often far beyond conventional templates—where susceptibility‐based preoperative maps were translated directly into intraoperative SLN retrieval [52].

Beyond pelvic staging, magnetic tracers enable topology‐resolved SLN/TDLN mapping across head‐and‐neck, gastrointestinal, and cutaneous lymphatic networks, where conventional tracers are limited by access, background signal, or template assumptions. For example, in early oral cancer, SPION‐enhanced MR lymphography enabled rapid and high‐yield SLN mapping—outperforming CT lymphography in both detection rate and nodal enumeration—by generating early susceptibility signals that delineated SLNs within minutes while later imaging visualized downstream drainage [53]. Similarly, in a 10‐patient pilot study of oral cancer, a fully magnetic SLNB workflow combining peritumoral SPION injection, preoperative MRI and handheld magnetometry enabled MRI‐based visualization and probe‐guided excision of magnetic SLNs. Complete magnetic SLNB was achieved in eight of ten patients, supporting the feasibility of this magnetic mapping route while indicating that it remains a small‐sample, early‐stage workflow requiring protocol optimization and larger validation [54]. In addition, radioisotope‐free approaches in clinically N0 oral cancer paired MR lymphography with cordless magnetometry to achieve precise SLN localization through mucosal lymphatics, with all mapped nodes retrieved magnetically [55]. Furthermore, in a preclinical head‐and‐neck model, mannose‐labelled magnetic iron oxide nanoparticles retained most lymphotropic iron within sentinel nodes and enabled level‐specific magnetic localization, suggesting a potential approach for more selective nodal mapping in anatomically complex basins [56]. Likewise, in a preclinical gastric model, the mannose‐labelled tracer FerroTrace—paired with ICG—enabled MRI‐, fluorescence‐ and magnetometer‐based localization of gastric SLNs, supporting the feasibility of more selective gastric SLN mapping [57]. In parallel, melanoma studies demonstrated that optimized SPION‐based MRI protocols rapidly reconstructed lymphatic drainage, identifying more candidate nodes than lymphoscintigraphy and enabling faithful intraoperative localization with magnetometers, while intracutaneous SPION delivery generated immediate susceptibility signals in first‐draining nodes [58]. Additionally, in apparent early‐stage endometrial cancer, cervicouterine SPION injection produced reliable pelvic SLN mapping with high bilateral detection, while intranodal magnetic accumulation enabled discrimination of metastatic involvement with favorable diagnostic performance and safety, extending topology‐resolved magnetic mapping into gynecologic basins [59].

### 3.2 High‐Fidelity Magnetic Signatures for Metastatic SLN/TDLN Discrimination

Building on topology reconstruction, a complementary strength of magnetic inorganic nanoparticles lies in their capacity to preserve high‐fidelity magnetic signatures that enable reliable discrimination between metastatic and benign SLNs/TDLNs. Stable intranodal iron retention, quantitative magnetometer readouts, and resistance to optical background or anatomical ambiguity collectively decouple nodal status assessment from subjective visual interpretation. In this context, magnetic signal fidelity itself becomes a decisive determinant of staging accuracy, particularly where conventional dyes or radioisotopes exhibit reduced contrast or reproducibility. For example, in an early clinical series of intermediate‐ and high‐risk prostate cancer, intraprostatic administration of superparamagnetic iron oxide nanoparticles enabled magnetometer‐guided SLN dissection, with magnetic SLNs identified in 19 of 20 patients. Metastases were detected in magnetic SLNs in four of the five node‐positive patients, supporting the feasibility and high detection rate of SPION‐guided SLN mapping [60]. Similarly, in breast cancer, Sienna+ SPION tracers accumulated robustly within sentinel nodes and achieved high patient‐ and node‐level concordance with technetium radiocolloids and blue dyes across multiple cohorts, reliably identifying metastatic involvement while remaining within predefined non‐inferiority margins [61]. In addition, multicenter paired comparisons confirmed that SPION‐guided localization matched technetium‐based mapping in SLN detection and metastatic concordance, while yielding comparable nodal retrieval and low postoperative complication rates, underscoring the inter‐institutional reproducibility of magnetic signal fidelity [62]. Furthermore, nonlinear magnetometry exploiting the field‐dependent harmonic response of SPIONs enabled highly sensitive SLN detection at low magnetic field amplitudes, selectively isolating true nodal signals from electromagnetic noise and metallic surgical instruments [63]. In parallel, handheld magnetometers engineered to operate at magnetic null points enabled sensitive transcutaneous and ex vivo detection of SPION‐laden nodes in breast cancer, with quantitative iron measurements supporting objective discrimination between sentinel nodes and surrounding tissue [64].

Beyond intraoperative assessment, magnetic signal fidelity also supports quantitative validation of metastatic involvement through ex vivo and multimodal approaches. For example, in an ex vivo colorectal SLN pilot study, magnetic nanoparticles enabled SLN selection after formalin fixation, with magnetically enriched nodes identifying isolated tumor cells with reported 100% sensitivity and accuracy and outperforming blue dye within this pilot setting [65]. Similarly, PET‐labeled magnetic nanoparticles combined preoperative PET‐based SLN identification with retained magnetic susceptibility for intraoperative magnetomotive ultrasound, where intranodal magnetic contrast closely matched ex vivo MRI quantification [66]. Moreover, in a preclinical mouse model, chelator‐free ^64^Cu–SPION hybrid nanoprobes unified quantitative PET sensitivity with MR anatomical precision, enabling PET/MRI visualization of lymphatic drainage and SLN accumulation for up to 24 h after administration [67]. Likewise, dextran‐coated magnetite nanoparticles labeled with ^99m^Tc functioned as dual radioactive–magnetic tracers, in which rapid lymphatic trafficking enabled lymphoscintigraphic resolution of nodal tiers while the magnetic core provided high‐resolution intraoperative detection in anatomically complex basins [68].

### 3.3 Temporal Decoupling and Workflow‐Adaptive Magnetic SLN/TDLN Mapping

Extending beyond topology reconstruction and signal‐fidelity–driven nodal discrimination, magnetic nanoparticles exhibit a distinctive advantage in temporal controllability and compatibility with complex clinical workflows. Owing to the stable ferrite magnetic core and prolonged intranodal retention of iron‐oxide nanoparticles, SLN/TDLN marking is no longer restricted to same‐day injection followed by immediate surgery. Instead, magnetic tracers can be flexibly embedded within neoadjuvant treatment courses, delayed surgical schedules, and multi‐step diagnostic–therapeutic pathways, allowing tracer kinetics to adapt to clinical practice rather than constraining operative timing. For example, in early breast cancer, preoperative outpatient administration of SPION results in stable sequestration within axillary sentinel lymph nodes for several weeks while preserving strong, magnetometer‐detectable signals at surgery, effectively relocating tracer injection outside the operating room and simplifying perioperative logistics [69]. Similarly, SPION injection 1–27 days before surgery maintained high sentinel node detection rates and yielded a slightly higher number of retrieved sentinel nodes than same‐day injection, supporting delayed scheduling flexibility and durable intranodal tracer retention [70]. In addition, in ductal carcinoma in situ (DCIS), peritumoral SPION injection at the time of initial breast surgery enables a “pre‐marking and selective retrieval” strategy, whereby SLNs are magnetically localized only if subsequent pathology confirms invasive disease, thereby sparing most patients unnecessary axillary surgery while preserving localization accuracy [71]. Furthermore, case‐based evidence demonstrates that SPION‐based magnetic labeling of tumor‐draining lymphatics can persist for more than 5 months, enabling reliable SLN/TDLN identification despite prolonged surgical delays and underscoring exceptional temporal stability [72].

Magnetic tracers also retain robust performance in treatment‐altered or multimodal clinical contexts. For example, in breast cancer patients undergoing neoadjuvant chemotherapy (NAC), evidence from a systematic review and meta‐analysis supports SPION as a workflow‐compatible tracer, with patient‐level detection comparable to radioisotope tracers and a higher number of SLNs identified in post‐treatment axillary staging [73]. Similarly, prospective tolerance data suggest that SPION‐based SLN mapping can be integrated into breast cancer pathways that include adjuvant radiotherapy, with no apparent increase in radiotherapy‐related toxicity observed after magnetic tracer use [74]. In parallel, combining SPION with magnetic tumor localizers such as Magseed enables a fully magnetic workflow that unifies non‐palpable tumor localization and SLN biopsy, delivering stable sentinel‐node detection and reliable margin control while substantially reducing reliance on nuclear medicine infrastructure [75]. Moreover, dose optimization further refines the clinical usability of magnetic nanotracing: the randomized SUNRISE study showed that 1 mL Sienna XP maintained non‐inferior SLN detection while reducing skin staining, supporting improved procedural tolerability within routine breast cancer SLN mapping [76] (Table 1). Magnetic nanotracers further extend SLN/TDLN mapping by combining lymphatic retention with preoperative imaging, radioisotope‐free intraoperative detection and greater scheduling flexibility. SPION‐based approaches are most strongly supported in breast cancer, particularly for delayed surgery and radiation‐free workflows, whereas applications in non‐breast tumors, multimodal magnetic navigation and newly developed detection devices are still supported by more limited feasibility or early validation studies. Thus, magnetic platforms bridge established clinical use and evolving workflow innovation.

**TABLE 1:** Magnetic inorganic nanoparticles enabling pathway‐based, high‐fidelity and temporally flexible SLN/TDLN mapping.

Type	Imaging principle	Clinical application	Performance in detection	Ref.
SPIONs	SPION‐mediated lymphatic transport	Prostate cancer	100% diagnostic accuracy, 96.6% sensitivity, 95.6% specificity	[49]
SPIONs	MRI with SPION and magnetometer‐guided SLN detection	Prostate cancer	100% diagnostic rate, 85.7% sensitivity, 97.2% specificity	[52]
SPIONs	MRL with SPION	Early oral cancer	90% detection rate by CTL, 100% detection rate by MRL, 35 total SLNs detected by CTL	[53]
SPIONs	MRI with SPION, magnetic handheld probe for SLN detection	Oral cancer, neck dissection	80% success rate, 3 SLNs with metastases detected	[54]
SPIONs	MRL with SPION, handheld cordless magnetic probe for SLN detection	Early oral cancer, sentinel lymph node biopsy	100% detection rate, 73 total SLNs identified	[55]
ML‐MIONs	83% of iron retained in sentinel node and linear increase in tracer uptake	Head and neck cancers, SLN biopsy	High affinity for SLN	[56]
FerroTrace	Utilizing SPIONs to enhance signal contrast	Gastric cancer, lymphadenectomy	100% detection rate by MRI and magnetometer, FerroTrace accumulates 76 ± 30% of total iron in SLNs	[57]
SPIONs	Enhancing T1‐weighted	Melanoma	100% SLN detection rate, 54 SLNs detected by GO‐FAST‐MRI	[58]
SPIONs	Enhancing signal contrast in SLN mapping	Endometrial cancer	100% detection rate, 70% bilateral SLN detection, sensitivity of 80%, AUC 0.9 for metastatic SLN detection	[59]
SPIONs	Enabling lymph node localization and identification in real‐time	Prostate cancer	80% sensitivity per patient, 100% sensitivity per patient when at least one SLN detected, 56% sensitivity per node	[60]
Sienna+ SPION	Enabling real‐time SLN localization through magnetic signal detection	Breast cancer	High agreement for SLN detection (PABAK 0.94), strong agreement for malignant SLNs (PABAK 0.89)	[61]
SPIONs	Utilizing SPIONs to identify SLNs via magnetic signal localization	Breast cancer	98.1% SLN identification rate, 99.0% concordance per patient, 97.4% concordance per node	[62]
SPIONs	Using SPION with a limited 5 mT field amplitude to eliminate interference	Alternative to technetium‐based tracers	High mass sensitivity, eliminating interference from metallic instruments	[63]
SPIONs	Basing on superparamagnetic properties of nanoparticles	Radioisotope‐free technique	10 mm longitudinal detection length for 140 µg iron, precise detection unaffected by ambient magnetic fields	[64]
SPIONs	Magnetometer‐based SLN localization post‐formalin fixation	Colorectal cancer	96% SLN identification rate with magnetic technique, 100% sensitivity and accuracy for occult metastases detection	[65]
^68^Ga‐labelled nanoparticles	MMUS based on the magnetic properties of nanoparticles	Pre‐operative PET and intra‐operative MMUS imaging	100% detection of SLNs by PET, 4 out of 6 SLNs detectable by MMUS	[66]
^64^Cu–SPION	Using radiotracer properties of ^64^Cu for PET	Preoperative planning and intraoperative guidance	97% labeling efficiency, specific accumulation in SLNs, clear SLN visualization	[67]
^99m^Tc–SPIONs	Dual radioactive and magnetic detection	Preoperative lymphoscintigraphy	98 ± 2% radiolabeling efficiency, 87.5% SLN identification by magnetometer probe	[68]
SPIONs	Basing on the superparamagnetic properties of SPIONs	Breast cancer	99% SLN identification rate	[69]
SPIONs	Allowing SLN identification via a magnetometer	Breast cancer	98.3% SLN detection rate for retro‐areolar, 97.4% for peri‐tumoral injections	[70]
SPIONs	Enabling SLN localization with minimal interference from surrounding tissue	Ductal carcinoma in situ	78.3% reduction in unnecessary SLND, 100% SLN detection with SPION plus blue dye	[71]
SPIONs	Enabling d‐SLNB with sustained activity in the lymph node for extended periods	D‐SLNB after breast‐conserving therapy	Sustaining effectiveness beyond typical 1‐month timeframe	[72]
SPIONs	Enabling high spatial resolution SLN detection using a magnetometer	Breast cancer	98.1% individual detection rate, 91.0% nodal detection rate	[73]
SPIONs	Magnetic detection based on SPION	Breast cancer	99.7% SLN identification rate	[74]
SPIONs	Magseed for tumor localization and SLN detection	Breast cancer	100% successful SNB, median of 2 SNs retrieved, no seed migration	[75]
Sienna XP	Enabling SLN localization through magnetic signal detection	Breast cancer	Non‐inferior SLN detection compared to ^99m^Tc, 100% concordance in SLN‐positive patients	[76]

## 4 Metal‐Derived Physicochemical Determinacy for Multimodal and Functionally Enriched SLN/TDLN Mapping

**FIGURE 3:**
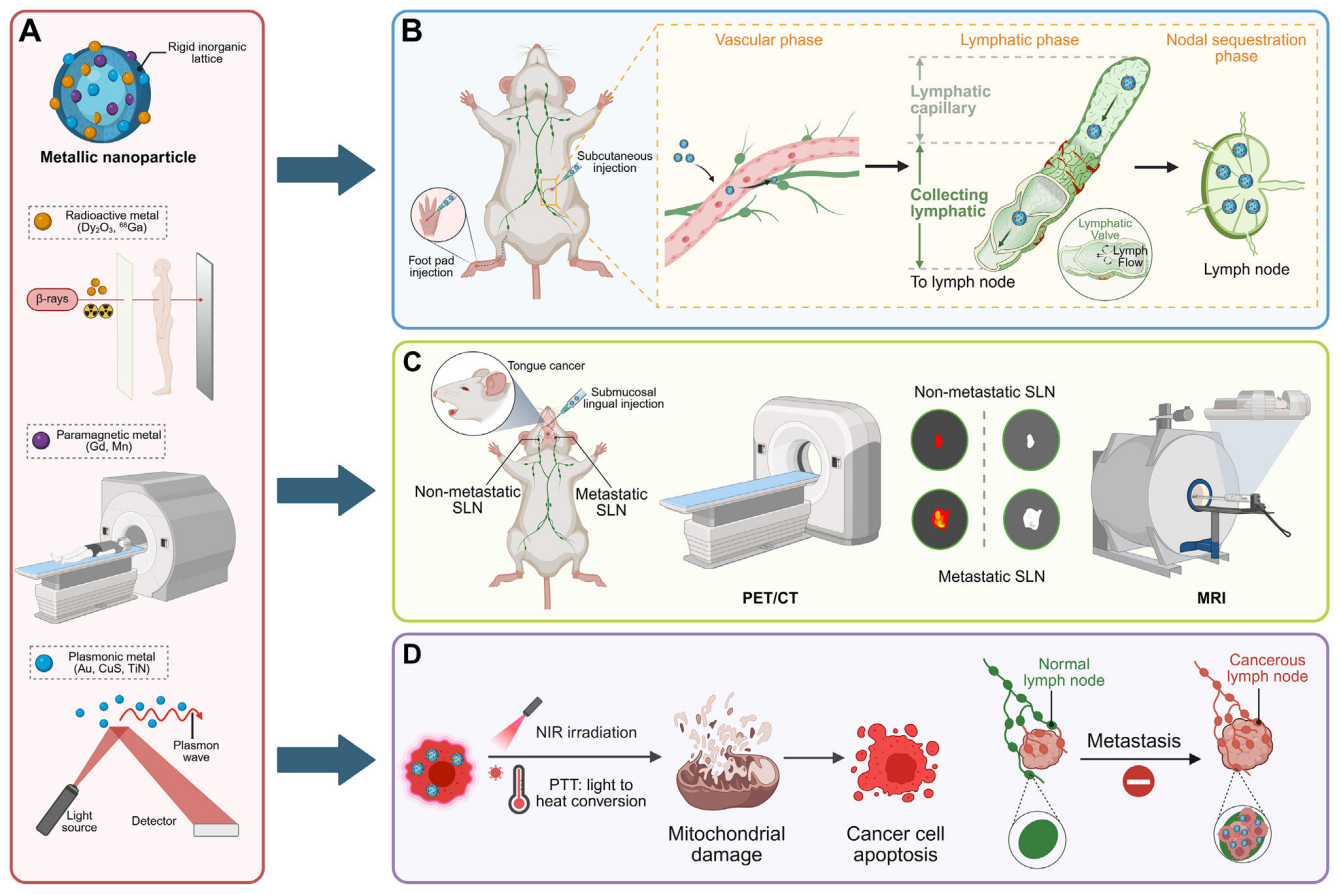
Metal‐based nanoparticles as intrinsic signal carriers for lymphatic tracking, nodal imaging, and local intervention. (A) Schematic illustration of a metal‐based nanoparticle featuring a rigid inorganic lattice as the structural core, within which radiometals, paramagnetic metals, and plasmonic domains are embedded. These metal‐derived components function as intrinsic signal sources, supporting radioactive decay, magnetic responsiveness, and plasmonic resonance. (B) Following local administration, nanoparticles preferentially enter lymphatic capillaries and are transported along collecting lymphatic vessels, resulting in stepwise accumulation within sentinel and downstream tumor‐draining lymph nodes. (C) Intrinsic PET/CT and MRI signals enable noninvasive visualization of lymph node status, allowing differentiation between non‐metastatic and metastatic SLNs based on signal distribution, integrity, and heterogeneity. (D) After nodal localization, plasmonically active nanoparticles permit NIR‐triggered photothermal conversion, inducing localized mitochondrial damage and cancer cell apoptosis within metastatic lymph nodes, thereby limiting further lymphatic spread.

Metal and metal‐oxide nanoparticles provide a structurally rigid and compositionally programmable inorganic platform for SLN/TDLN detection, distinguished by their stable signal sources, high atomic number elements, and well‐defined physicochemical contrast mechanisms [77]. Unlike soft organic or polymeric systems, these nanostructures embed radiometals, paramagnetic centers, or plasmonic components directly within crystalline lattices, enabling quantitative, multimodal lymphatic imaging with high spatial fidelity. Importantly, the deterministic signal behavior of metal‐based nanoplatforms supports not only robust nodal localization but also structure‐informed discrimination of metastatic involvement, and, in selected designs, localized nodal modulation following detection. Collectively, these properties position metal and metal‐oxide nanoparticles as a distinct class of lymphotropic probes that extend SLN/TDLN mapping from reliable localization toward functionally enriched nodal interrogation (Figure 3A).

### 4.1 Intrinsic Metal‐Derived Multimodal SLN/TDLN Localization

A defining strength of metal‐based nanoplatforms lies in their ability to deliver stable, quantitative, and multimodal signals for high‐fidelity SLN/TDLN localization. For example, gadolinium‐loaded, polyethylenimine‐entrapped gold nanoparticles exemplify this principle by coupling rigid nanostructure–mediated lymphatic transport with persistent CT/MR contrast. After submucosal injection, these nanoprobes migrate through afferent lymphatic vessels and accumulate selectively within lingual sentinel lymph nodes, where black staining colocalizes with CT/MR enhancement and supports 100% SLN identification in the rabbit model [78]. Complementing this approach, dendrimer‐entrapped gold nanoparticles achieve sustained CT enhancement of cervical sentinel nodes over a 72‐h window, with prolonged intranodal retention supporting accurate anatomical localization during delayed surgical navigation [79]. Extending this multimodal strategy, porphyrin‐based ^68^Ga‐F127‐TAPP/TCPP(Mn) nanoparticles integrate PET radiolabeling, enhanced MRI contrast, and intrinsic nodal staining within a single construct. After lymphatic migration, these nanoparticles accumulate differentially in normal and metastatic lymph nodes, producing PET/MR readouts that distinguish nodal metastatic status while also providing dark‐brown staining for potential visual mapping [80]. Together, these examples illustrate how rigid inorganic architectures translate compositional programmability into highly deterministic SLN/TDLN detection across complementary imaging modalities (Figure 3B).

### 4.2 Microstructural and Pathological SLN/TDLN Discrimination Enabled by Metal Signals

Beyond nodal localization, selected metal‐based nanoplatforms leverage their sensitivity to tissue microstructure and contrast heterogeneity to discriminate metastatic from non‐metastatic SLNs. Gd–Au PENPs illustrate this capability by revealing tumor‐induced disruption of nodal architecture: whereas benign SLNs display uniform enhancement and preserved contours, metastatic nodes exhibit irregular morphology, heterogeneous signal distribution, and characteristic filling defects reflecting microanatomical collapse. These contrast patterns enabled non‐invasive identification of metastatic SLNs in a rabbit tongue VX2 model, with 100% sensitivity and negative predictive value, although specificity was more limited [81] (Figure 3C). Similarly, Pluronic‐stabilized ICG/TCPP(Mn) nanoparticles provide sufficient imaging sensitivity to resolve not only metastatic involvement but also differences between micro‐ and macrometastatic disease. The combination of strong intranodal fluorescence and Mn‐driven MRI contrast allows signal intensity and spatial distribution to reflect metastatic burden, supporting both preoperative characterization and intraoperative decision‐making [82]. Collectively, these systems move metal‐based nanodetection beyond simple SLN visualization toward pathology‐informed interpretation, in which signal morphology, intensity, and spatial patterning provide insight into nodal metastatic status.

### 4.3 Detection‐Coupled Nodal Intervention Driven by Metal Nanoplatforms

A distinctive feature of several metal and metal‐oxide nanoplatforms is their capacity to couple SLN/TDLN detection with localized nodal intervention within the same construct. Intrinsically radiolabeled dysprosium oxide nanoparticles exemplify this integration by enabling high‐contrast SLN mapping after lymphatic uptake and nodal retention, while their ^166^Dy/^166^Ho β‐emitting generator design suggests potential for selective radiotherapeutic intervention after detection [83]. Photothermally active metal nanostructures further illustrate detection‐guided intervention. Titanium nitride nanobipyramids exploit strong plasmonic scattering for optical SLN delineation across visible and near‐infrared windows, while subsequent NIR‐II irradiation converts localized nanoparticle accumulation into efficient heat generation, enabling selective ablation of metastatic lymph nodes and suppression of downstream metastasis [84] (Figure 3D). Likewise, galectin‐1–targeted TDG@PEG@MnPb‐IR820 nanoparticles combine MRI/NIR detection with targeted retention in a cervical‐cancer lymph‐node metastasis model, allowing imaging‐guided photothermal activation of metastatic tumor‐draining nodes [85]. Together, these platforms demonstrate how metal‐based nanoparticles can integrate SLN/TDLN detection with spatially confined nodal intervention, establishing a functional continuum from localization to intervention. Metal and metal‐oxide nanoplatforms expand SLN/TDLN mapping from a different angle. Their appeal is rooted in the intrinsic physical properties of inorganic structures, which can incorporate radiometal, paramagnetic or plasmonic functions into a single lymphotropic probe. These platforms have therefore broadened the conceptual scope of nodal imaging toward multimodal localization, metastatic‐node interrogation and detection‐guided local intervention. However, most examples remain supported by animal studies or early proof‐of‐concept experiments, and their translational value will require validation against established clinical tracers.

## 5 Other Rigid Inorganic Nanoplatforms Governed by Intrinsic Material Properties for SLN/TDLN Mapping

Beyond magnetic and metal‐derived systems, a diverse group of structurally rigid inorganic nanoparticles—encompassing silica, metal sulfides, and rare‐earth–based nanocrystals—enable SLN/TDLN mapping through geometry‐ and material‐intrinsic signal mechanisms.

### 5.1 Silica‐Based Nanoparticles for Size‐Governed SLN/TDLN Mapping

Silica‐based nanoparticles constitute a class of inorganic lymphatic tracers defined by a rigid SiO_2_ core and precise control over particle size [86]. Their lymphatic transport and nodal distribution are governed predominantly by geometric parameters rather than intrinsic physical signal properties, enabling direct interrogation of size‐dependent access to sentinel and tumor‐draining lymph nodes.

**FIGURE 4:**
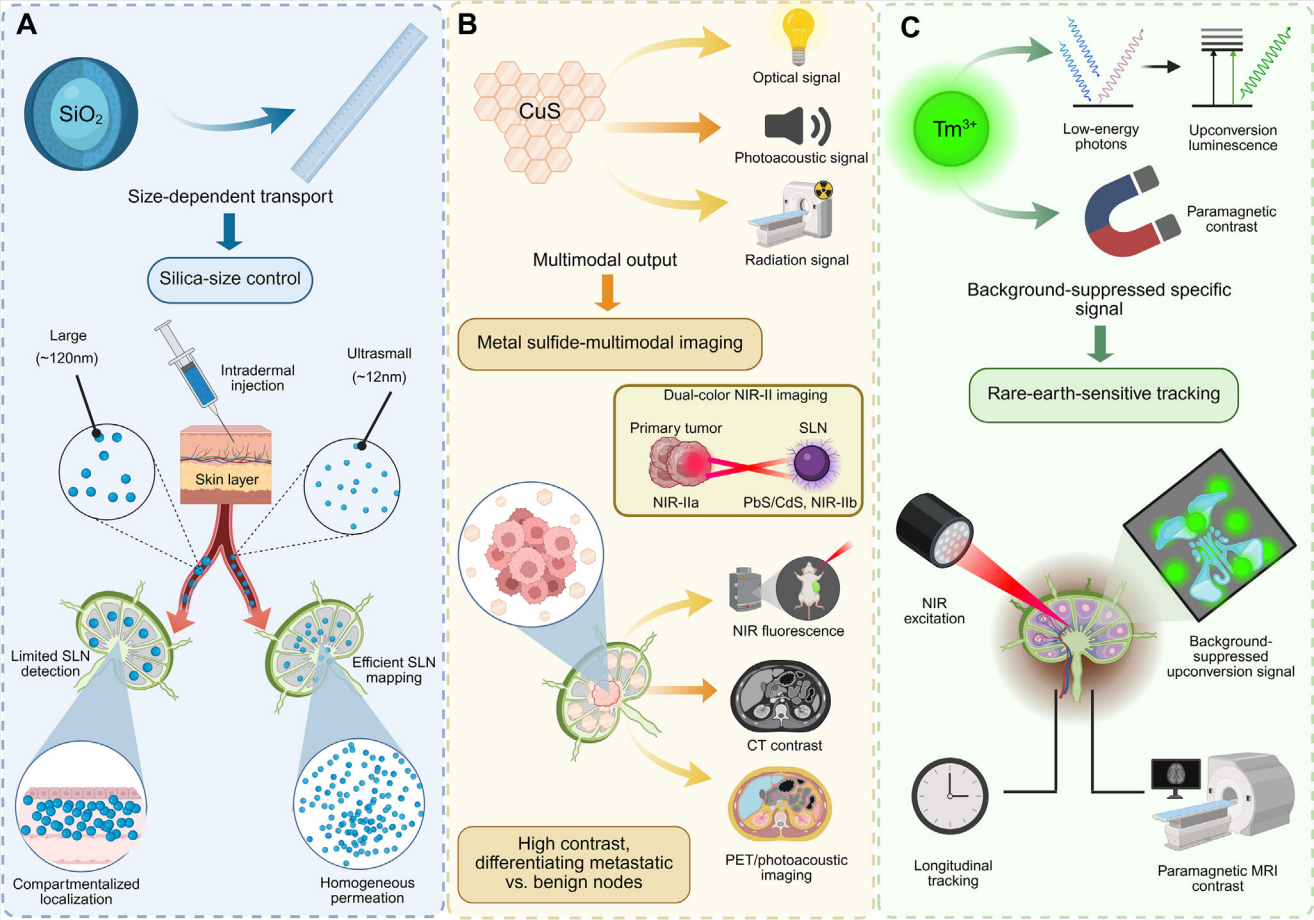
Rigid inorganic nanoplatforms for SLN/TDLN mapping. (A) Silica‐based nanoparticles: Size governs lymphatic transport. Ultrasmall particles (∼12 nm) enable efficient SLN mapping, while large particles (∼120 nm) exhibit restricted access. (B) Metal sulfide nanoplatforms: Intrinsic properties enable high‐sensitivity, multimodal imaging (optical, photoacoustic, PET). This supports advanced applications like dual‐color NIR‐II imaging for simultaneous tumor and SLN visualization. (C) Rare‐earth‐based nanoparticles: Lanthanide‐doping provides background‐suppressed upconversion luminescence for sensitive, longitudinal SLN tracking, complemented by paramagnetic contrast for MRI.

Ultrasmall core–shell silica nanoparticles illustrate this principle by coupling a SiO_2_ framework with molecular targeting and fluorescence for sensitive SLN detection. In head and neck melanoma, integrin‐targeted core–shell silica nanoparticles (cRGDY–PEG–Cy5.5) accumulated selectively in sentinel lymph nodes after intradermal injection, showing high concordance with technetium‐defined SLNs and signal‐to‐background ratios sufficient for transcutaneous and intraoperative fluorescence guidance [87]. Complementary size‐controlled silica nanoparticles further defined the role of particle dimension in lymphatic trafficking. Monodisperse silica particles spanning 7–120 nm demonstrated that ultrasmall nanoparticles (∼12 nm) rapidly entered afferent lymphatics and homogeneously permeated SLNs, whereas larger particles (∼120 nm) exhibited restricted transport and compartmentalized intranodal localization, limiting early SLN detection [88] (Figure 4A). Together, these studies establish particle size as a primary determinant of efficient SLN/TDLN mapping using silica nanoplatforms.

### 5.2 Metal Sulfide Nanoparticles for Signal‐Intensive SLN/TDLN Mapping

Metal sulfide nanoparticles are defined by a crystalline metal–sulfur (M–S) core that confers strong optical, photoacoustic, or radiometric signal output for lymphatic imaging [89]. Unlike metallic or oxide‐based nanomaterials, their utility in SLN/TDLN mapping arises from intrinsic semiconductor or plasmonic properties rather than magnetic or high‐atomic‐number contrast. These signal characteristics enable sensitive detection of sentinel and tumor‐draining lymph nodes through multimodal imaging approaches.

Copper sulfide nanoparticles exemplify this strategy by integrating lymphatic transport with multimodal signal generation for precise SLN/TDLN detection. RGD‐functionalized CuS nanostructures rapidly drained from primary gastric tumors to sentinel lymph nodes, where αvβ3‐integrin–mediated uptake in metastatic cells generated enhanced near‐infrared fluorescence and CT contrast, enabling discrimination between metastatic and benign nodes [90] (Figure 4B). In a complementary design, radiolabeled ^64^CuS nanoparticles enabled dual PET–photoacoustic mapping of axillary SLNs, combining persistent PET signals for preoperative localization with high‐resolution photoacoustic imaging for intraoperative identification of first‐echelon nodes [91]. Semiconductor quantum dots further extend metal sulfide nanoplatforms into the NIR‐II regime for SLN visualization. PbS/CdS core–shell quantum dots accumulated selectively in cancer‐invaded sentinel lymph nodes, producing ultrabright NIR‐IIb fluorescence with minimal tissue scattering and autofluorescence. When paired with a spectrally distinct NIR‐IIa tumor probe, this system enabled simultaneous visualization of primary tumors and metastatic SLNs, supporting precise intraoperative identification of tumor‐draining lymphatic basins [92]. Together, these studies illustrate how metal sulfide nanoparticles exploit intrinsic semiconductor properties to achieve high‐sensitivity SLN/TDLN mapping across optical, acoustic, and nuclear imaging modalities.

### 5.3 Rare‐Earth–Based Inorganic Nanoparticles for SLN/TDLN Mapping

Rare‐earth–based inorganic nanoparticles are defined by crystalline lattices dominated by lanthanide elements, endowing them with intrinsic optical or paramagnetic contrast for lymphatic imaging. Their role in SLN/TDLN mapping relies on materials‐driven signal generation rather than magnetic susceptibility or high‐atomic‐number attenuation. These properties enable stable and multimodal visualization of tumor‐draining lymph nodes.

Tm^3+^‐doped upconverting nanoparticles illustrate this class by enabling non‐invasive SLN detection through NIR‐to‐NIR upconversion luminescence. After peripheral administration, these UCNPs entered afferent lymphatics and accumulated in sentinel lymph nodes, producing background‐suppressed signals that allowed longitudinal SLN tracking over extended periods [93] (Figure 4C). Complementarily, GdF_3_ nanoparticles exploit lanthanide‐based paramagnetism to support multimodal SLN/TDLN mapping. Biomineralized GdF_3_@transferrin nanoparticles accumulated efficiently in sentinel lymph nodes and enabled combined MRI, CT, PET, and NIR imaging through integrated radiolabeling and fluorophore conjugation, providing precise localization of tumor‐draining lymphatic basins [94]. Rigid inorganic nanoparticles leverage intrinsic material properties for SLN/TDLN mapping, including size‐dependent transport, semiconductor‐based signals, and lanthanide‐derived contrast. Selected silica probes have been evaluated in early clinical studies, while most metal sulfide and rare‐earth platforms remain in preclinical or proof‐of‐concept stages. Overall, these studies provide mechanistic insight into lymphatic distribution, signal modulation, and functional enhancement, highlighting that standardized clinical validation is still needed for most platforms.

## 6 Polymeric and Organic Nanoparticles for Programmable and Functionally Adaptive SLN/TDLN Mapping

Polymeric and organic nanoparticles constitute a highly versatile class of lymphotropic probes for SLN and TDLN detection, enabled by their synthetic flexibility, programmable architectures, and capacity for biologically responsive signal control. Rather than relying on intrinsic physical contrast, these systems leverage chemical composition, supramolecular assembly, and microenvironment‐sensitive activation to regulate lymphatic trafficking, intranodal retention, and signal generation. Such design freedom allows polymeric nanoplatforms to address key challenges in lymphatic mapping, including background suppression, metastatic specificity, and long‐term nodal marking, while maintaining favorable biocompatibility profiles.

### 6.1 High‐Resolution SLN Mapping Through Constitutive Optical or Acoustic Contrast

**FIGURE 5:**
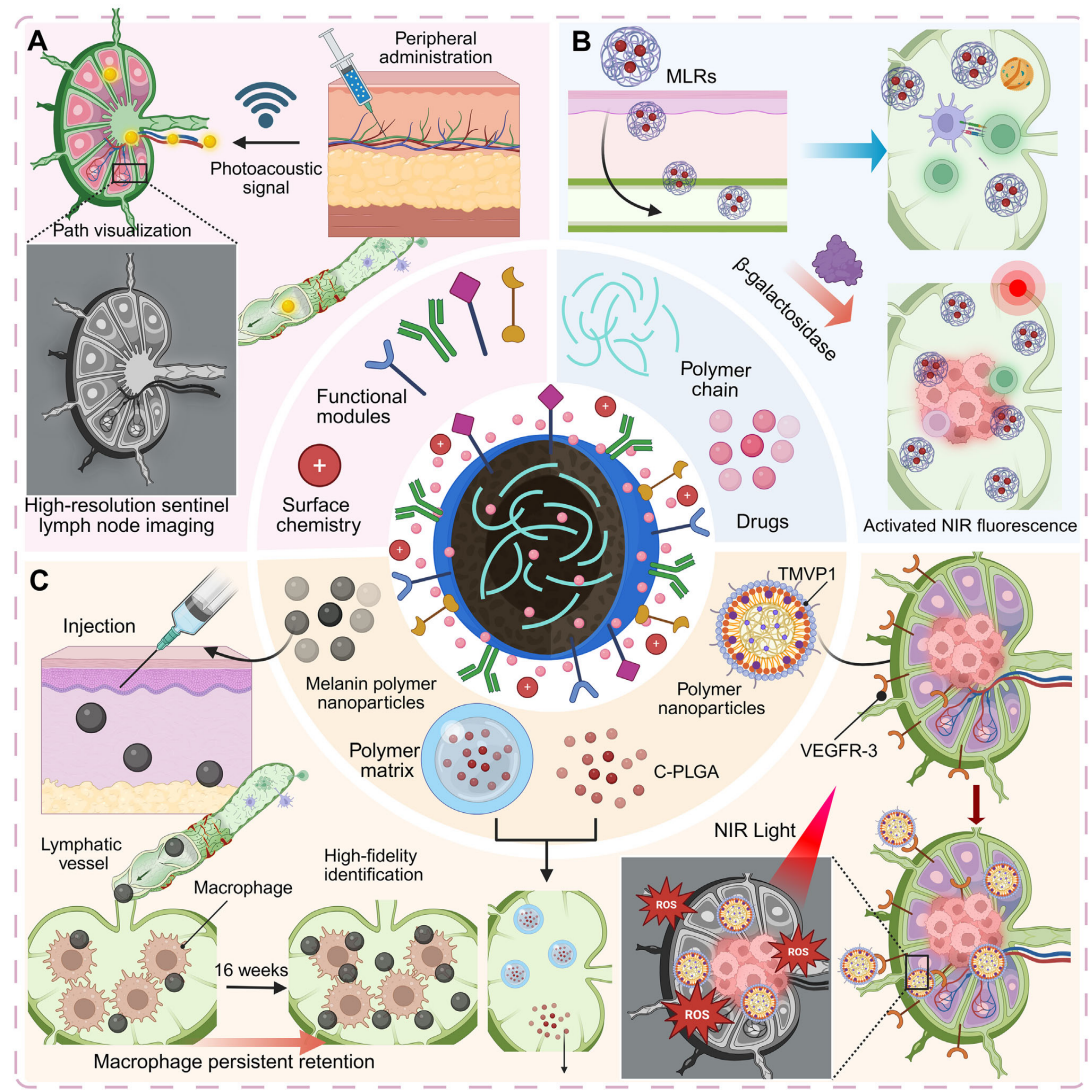
Nanoparticles for sentinel lymph node imaging and precision therapy. (A) Conjugated oligomer nanoparticles produce high‐contrast photoacoustic signals after peripheral administration, enabling non‐ionizing, real‐time visualization of sentinel lymph node drainage pathways and photothermal ablation of metastatic lymph nodes. (B) MLRs efficiently drain into the lymphatic system and activate near‐infrared fluorescence only after β‐galactosidase‐mediated decaging in metastatic gastric cancer cells. (C) Melanin‐loaded polymeric nanoparticles provide a stable, endogenous pigment‐based strategy for sentinel lymph node labeling, with persistent retention by macrophages and high‐fidelity node identification up to 16 weeks post‐injection; Carbon‐loaded PLGA particles encapsulate carbon in a biodegradable polymer matrix, enhancing long‐term lymphatic labeling with more stable nodal retention than traditional carbon suspensions; TMVP1‐modified polymeric nanoparticles target VEGFR‐3 for selective accumulation in metastatic lymph nodes, enabling image‐guided photothermal‐photodynamic combinatorial ablation.

Polymeric nanoparticles with constitutive optical or acoustic contrast provide a direct means to visualize lymphatic drainage and delineate SLN topology with high spatial resolution. By tailoring conjugated backbones, emission properties, and particle size, these systems achieve efficient lymphatic entry and stable accumulation in first‐echelon nodes, enabling real‐time SLN mapping without reliance on radioactive tracers. For example, conjugated‐oligomer nanoparticles with strong near‐infrared absorbance generate high‐contrast photoacoustic signals after peripheral administration, enabling non‐ionizing, real‐time visualization of SLN drainage pathways; their PA activity also permits localized photothermal ablation of metastatic cells within mapped nodes [95] (Figure 5A). Aggregation‐induced emission nanoparticles constructed from benzodithiadiazole‐based luminogens similarly combine robust lymphatic trafficking with dual photoacoustic and NIR‐II fluorescence output, allowing sensitive detection of SLNs—including nodes as small as ∼1 mm and located distantly from the primary lesion—and accurate intraoperative guidance in neuroendocrine neoplasms [96]. In parallel, a donor–acceptor conjugated polymer nanoprobe (CDT‐TT) introduces a fluorescence‐free Raman imaging strategy, producing a sharp and isolated vibrational signature that enables precise delineation of in situ SLN boundaries without tissue autofluorescence interference, thereby extending polymeric SLN mapping into label‐free optical domains [97].

### 6.2 Signal‐Activatable Discrimination of Metastatic SLNs

A defining advantage of polymeric nanoplatforms lies in their ability to decouple lymphatic transport from signal activation, enabling selective fluorescence or contrast generation within tumor‐involved lymph nodes. By remaining optically silent during lymphatic migration and activating only in response to tumor‐associated enzymes or intracellular processing, these systems enhance metastatic specificity while minimizing background signal from benign nodes. Representative of this strategy, metastatic‐lymph‐node reporters (MLRs) are self‐assembled nanostructures that drain efficiently into lymphatics but activate near‐infrared fluorescence only upon β‐galactosidase–mediated uncaging in metastatic gastric cancer cells. This mechanism yields high‐contrast discrimination of micro‐ and macrometastatic SLNs—including skip metastases—with sensitivity approaching flow cytometry in both animal models and patient specimens [98] (Figure 5B). Similarly, fluorophore–quencher–engineered nanoparticles assembled from dendrimers, polyethyleneimine, and γ‐polyglutamic acid remain optically silent until lysosomal uptake within draining nodes triggers FRET dequenching, confining fluorescence generation to SLNs and sharpening detection specificity [99]. Together, these activatable polymeric systems illustrate how biochemical responsiveness can transform SLN visualization into a functionally informative readout of metastatic involvement.

### 6.3 Durable Nodal Marking, Targeting, and Workflow Adaptability

Beyond immediate detection, polymeric nanoparticles have been engineered to support durable SLN/TDLN marking and robust performance across complex clinical workflows, including neoadjuvant therapy and delayed surgery. By exploiting controlled degradation, surface charge modulation, and pigment encapsulation, these platforms achieve prolonged intranodal retention while maintaining biological compatibility. Notably, melanin‐loaded polymeric nanoparticles provide a stable and endogenous pigment‐based strategy for SLN tattooing, enabling high‐fidelity identification of marked nodes even up to 16 weeks after injection through persistent macrophage‐associated retention [100]. Carbon‐loaded PLGA particles further strengthen long‐term lymphatic marking by encapsulating carbon within biodegradable polymer matrices, yielding more consistent nodal retention and resistance to lymphatic remodeling than conventional carbon suspensions [101]. In addition, mannan‐based copolymers bearing dual MR and fluorescence reporters exploit DC‐SIGN–mediated macrophage targeting to accumulate selectively in SLNs and downstream TDLNs, supporting non‐invasive mapping of metastatic pathways [102]. Finally, TMVP1‐modified polymer nanoparticles illustrate how molecular targeting can be integrated with SLN detection, using VEGFR‐3 recognition to achieve selective accumulation in metastatic nodes and enabling imaging‐guided photothermal–photodynamic ablation [103] (Figure 5C). Collectively, these examples highlight how polymer chemistry enables SLN/TDLN detection strategies that are not only sensitive and specific, but also adaptable to real‐world clinical timelines and interventions (Table 2). Polymeric nanoparticles exhibit a high degree of programmability and functional adaptability for SLN/TDLN mapping, regulating lymphatic transport, intranodal retention, and signal output through chemical composition, supramolecular assembly, and microenvironment‐sensitive activation. These capabilities enable signal activation, enhanced metastatic specificity, durable nodal marking, and multimodal imaging. In preclinical animal models and in vitro experiments, such platforms have demonstrated sensitive, specific, and persistent lymph node visualization while providing mechanistic and functional insight for further optimization. Nevertheless, the validation to date remains largely preclinical, and systematic studies with standardized protocols will be required to assess their feasibility and translational potential in clinical settings.

**TABLE 2:** Bio‐derived and biomimetic nanoparticles for biologically driven SLN/TDLN mapping.

Type	Mechanism	Clinical application	Advantages	Performance in detection	Ref.
Gadolinium lipid nanoparticles	Basing on gadolinium‐loaded nanoparticles	Tumor‐induced alterations in lymph drainage	Improving specificity and sensitivity	Preferential uptaking in TDLN	[104]
Gadolinium lipid nanoparticles	Enhancing MRI sensitivity	Melanoma TDLN detection	Higher specificity and sensitivity for detecting SLNs	Selective uptaking in TDLN	[105]
MSA + NBB	^99m^Tc‐MSA‐NBB conjugate binds to SLNs	Multimodal imaging (SPECT/CT, fluorescence)	Sustaining for 2 h, high sensitivity	^99m^Tc‐MSA‐NBB conjugate showed high accumulation	[106]
nTAL	Tumor‐specific conversion of MAL to fluorescent PPIX	Metastatic status during surgery	Early detection of metastasis, non‐invasive	Differentiation metastatic from benign LNs	[107]
SiNPs@EXO	Tumor‐homing exosomes for targeting metastatic SLNs	Lymphatic metastasis prediction	Stable long‐term tracking	Peaks within 0.5h and remains for up to 3h	[108]
MLT‐HA‐HPPS	Electrostatic interaction for loading melittin on HA‐HPPS	Breast cancer	Dual targeting to both breast cancer and SLNs	81.3% inhibition of primary tumor growth	[109]
LF‐NP@PEG	Surface modification of LF‐NPs with PEG	SLN detection	Improving size uniformity, reducing batch variation	Greater cellular uptake with LF‐NP@PEG	[110]
^86^Re/^188^Re‐LNP	Targeting delivery of β‐emitting radionuclides	Postlumpectomy treatment	High absorbed dose to lumpectomy cavity and SLNs	Effective dose delivery to 2‐ and 5‐mm depths	[111]
BSM‐ liposome	BSM nanoparticles absorb NIR light	Cancer photothermal therapy	Non‐toxic, easy to obtain from black sesame seeds	Photothermal therapy‐induced tumor growth suppression	[112]
Dex‐MBA‐PAA‐Gd	High‐relaxivity T1‐weighted MRI contrast agent	SLN mapping, MRI imaging	High longitudinal relaxivity (r1) and biosafety	Effective SLN visualization and accurate positioning	[113]

## 7 Bio‐Derived and Biomimetic Nanoparticles for Biologically Driven SLN/TDLN Mapping

Bio‐derived and biomimetic nanoparticles exploit endogenous materials, biological recognition motifs, or cell‐mimicking architectures to achieve efficient lymphatic trafficking and selective nodal retention. In contrast to purely synthetic or inorganic systems, these platforms leverage physiological transport routes, immune cell interactions, and tumor‐associated enzymatic or receptor cues to support SLN/TDLN mapping with high biocompatibility. By embedding detection functions within biologically familiar carriers, biomimetic nanotechnologies offer a complementary paradigm for lymphatic navigation that aligns naturally with complex tissue environments and clinically relevant workflows.

### 7.1 Functional Lymphatic Pathway Reconstruction and TDLN Topology

A subset of bio‐derived nanoplatforms demonstrates particular strength in reconstructing tumor‐altered lymphatic drainage pathways and resolving TDLN topology beyond canonical anatomical expectations. Rather than merely highlighting first‐accessible nodes, these systems emphasize functional drainage hierarchies by exploiting differential transport, compartmentalization, and retention within tumor‐conditioned lymphatic networks. For example, gadolinium‐loaded lipid nanoparticles preferentially accumulated in tumor‐draining popliteal lymph nodes after subcutaneous administration, where extension of MR contrast from the cortex into the medulla delineated tumor‐induced remodeling of lymphatic flow, while uninvolved nodes restricted signal to the cortex and second‐tier nodes exhibited reduced uptake [104]. Similarly, biomimetic hybrid ICG–^99m^Tc nanocolloids exploited physiological lymphatic transport to enable direct mapping of sentinel drainage in muscle‐invasive bladder cancer, with concordant radiographic and near‐infrared signals revealing tumor‐draining pathways extending beyond standard pelvic dissection fields [105]. In addition, a ^99m^Tc‐labelled mannosylated albumin nanocarrier conjugated with blue dye rapidly concentrated within first‐draining lymph nodes via macrophage‐mediated uptake, providing durable multimodal visualization that clarified the hierarchical organization of SLNs and downstream nodal stations [106].

### 7.2 Metastasis‐Selective SLN/TDLN Discrimination

Beyond pathway reconstruction, several biomimetic nanotechnologies introduce tumor‐dependent signal activation or selective nodal enrichment mechanisms that enable discrimination between benign and metastatic SLNs based on biological specificity rather than passive lymphatic filtration alone. A representative example is the tumor‐activatable liposomal nanoprobe (nTAL), which drained efficiently to SLNs but remained optically silent until tumor‐specific enzymatic conversion of methyl aminolevulinate to fluorescent protoporphyrin IX occurred within metastatic nodes, enabling real‐time identification of early nodal involvement. The same nTAL platform further showed that PPIX fluorescence correlated with tumor cell infiltration in lymph nodes, supporting biologically activated assessment of metastatic burden within SLNs [107]. Similarly, silicon‐nanoparticle–loaded tumor‐derived exosomes combined durable inorganic fluorescence with the intrinsic homing capacity of cancer vesicles, resulting in rapid and persistent accumulation within metastatic SLNs and enabling early discrimination of nodal metastasis with minimal background signal [108]. In parallel, hyaluronic‐acid–conjugated HDL‐mimetic nanoparticles loaded with melittin trafficked from primary tumors into draining SLNs, where selective accumulation supported fluorescence‐guided identification of metastatic nodes alongside substantial suppression of nodal tumor burden [109].

### 7.3 Workflow Integration, Durability, and Multimodal Clinical Utility

An additional advantage of bio‐derived and biomimetic nanoparticles lies in their compatibility with clinically realistic workflows, including durable nodal marking, multimodal navigation, and the integration of detection with localized therapy. For instance, PEG‐modified lactoferrin nanoparticles improved SLN uptake relative to unmodified lactoferrin nanoparticles and produced detectable scintigraphic signals in a rat footpad model, although their retention and overall in vivo distribution still required further optimization [110]. Moreover, dosimetric modeling of liposome‐encapsulated β‐emitting radionuclides (^186^Re/^188^Re) suggested that lymphatic drainage from the post‐lumpectomy cavity could theoretically deliver focal therapeutic radiation to draining SLNs while limiting dose to surrounding tissues [111]. Naturally derived black sesame melanin nanosheets, stabilized by liposomal encapsulation, passively delineated SLNs via intrinsic pigmentation and separately enabled near‐infrared–driven photothermal suppression of tumor growth in an in vivo cancer model [112]. In addition, dextran–gadolinium nanoparticles engineered with multivalent chelation architectures generated bright and stable MR contrast in draining nodes, providing radiation‐free anatomical localization of SLNs with favorable biosafety profiles [113]. Bio‐derived and biomimetic nanoparticles add another layer of design flexibility by incorporating physiological transport, immune cell uptake, tumor‐associated activation or vesicle‐mediated homing into lymphatic mapping. This group spans a broad developmental spectrum, from clinically adjacent hybrid tracers to preclinical imaging probes, therapeutic proof‐of‐concept systems and modelling‐based designs. Its current value lies in showing how biological recognition and endogenous trafficking can be engineered to refine SLN/TDLN navigation, while standardized clinical workflows remain to be established for many of these platforms.

## 8 Challenges and Future Perspectives

**FIGURE 6:**
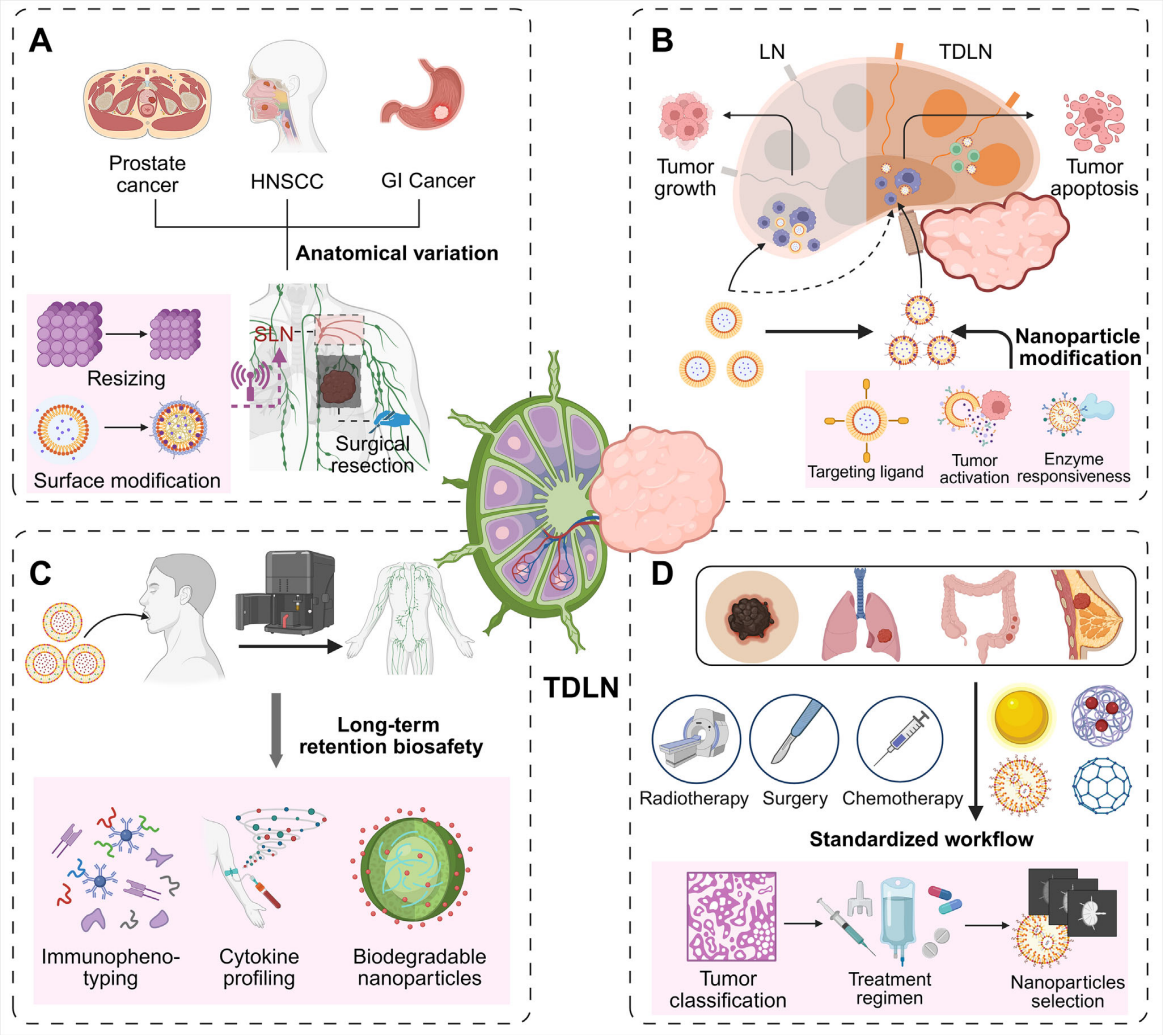
Major challenges and future directions for nanoparticle‐enabled SLN and TDLN mapping. (A) Anatomically complex tumors, including prostate cancer, HNSCC, and gastrointestinal malignancies, exhibit substantial interpatient variability in lymphatic topology as a result of tumor‐induced lymphangiogenesis, pathway remodeling, and collateral drainage. Rational control of nanoparticle size and surface properties, together with pathway‐aware surgical planning, may improve identification of biologically relevant first‐echelon nodes. (B) Because nodal tracer accumulation does not necessarily reflect true metastatic burden, next‐generation nanoplatforms are being engineered with targeting ligands and tumor‐ or enzyme‐responsive activation mechanisms to enhance selective visualization of biologically relevant tumor‐draining nodes. (C) Prolonged nanoparticle retention within lymph nodes raises biosafety concerns that extend beyond acute toxicity, underscoring the need for long‐term immunophenotyping, cytokine profiling, and the development of biodegradable or clearance‐tunable nanomaterials. (D) Broad clinical translation will require standardized, pathway‐aware, and protocol‐driven workflows that align nanoparticle selection with tumor type, treatment sequence, and multimodal management, thereby enabling reproducible integration with radiotherapy, surgery, and chemotherapy across institutions.

Despite substantial advances in nanoparticle‐enabled visualization of SLN/TDLN, several unresolved challenges continue to constrain their interpretability and clinical generalizability. These limitations arise not simply from insufficient signal intensity, but from the intrinsically dynamic, heterogeneous, and treatment‐responsive nature of tumor‐associated lymphatic systems [114, 115]. As SLN/TDLN mapping expands from intraoperative localization toward pathway‐level interrogation and longitudinal clinical integration, ensuring that nanoparticle‐derived signals faithfully correspond to biologically relevant first‐echelon nodes, metastatic status, and long‐term safety remains a central unmet need (Figure 6A).

Tumor‐induced lymphangiogenesis, lymphatic pathway remodeling, and the emergence of collateral drainage networks introduce substantial interpatient variability in lymphatic topology, particularly in anatomically complex malignancies such as prostate, head‐and‐neck, and gastrointestinal cancers [116]. In these contexts, functionally dominant sentinel lymph nodes frequently deviate from canonical anatomical expectations and may reside outside standard dissection templates. Although lymphotropic nanoparticles can reliably access and label early‐draining nodes, the nodes that accumulate tracer most prominently do not necessarily correspond to the biologically decisive first‐echelon basin that governs metastatic dissemination. As a result, tracer‐defined SLNs may sometimes reflect accessibility rather than true hierarchical position within the tumor‐draining lymphatic network [117, 118]. Addressing this challenge requires a conceptual shift from static, anatomy‐based definitions of SLNs toward a functional and dynamic framework that captures lymphatic flow patterns, temporal tracer kinetics, and nodal interconnectivity. Future nanoparticle‐enabled strategies should therefore emphasize pathway reconstruction rather than simple node enumeration, integrating time‐resolved imaging, quantitative signal analysis, and computational modelling to infer nodal hierarchy on a patient‐specific basis. Rational nanoparticle design—incorporating tunable size, surface chemistry, and receptor affinity—may further enhance discrimination between transient drainage nodes and bona fide first‐echelon SLNs. Such approaches will be essential for improving the biological fidelity of SLN/TDLN mapping and for ensuring that nodal detection more accurately reflects metastatic risk rather than anatomical convenience (Figure 6B).

Most nanoparticles currently employed for SLN/TDLN detection are preferentially sequestered by nodal macrophages, subcapsular sinus cells, or other phagocytic compartments following lymphatic drainage [11, 119]. This uptake behavior confers strong nodal contrast and prolonged intranodal retention, which are advantageous for localization and workflow flexibility. However, it also introduces fundamental ambiguity in signal origin, as nanoparticle accumulation predominantly reflects lymphatic filtration efficiency and immune cell engagement rather than direct association with tumor cells [120]. As a consequence, nodal signal intensity often correlates poorly with true metastatic burden, particularly in scenarios involving micrometastatic disease, scattered tumor cell clusters, or residual disease following neoadjuvant therapy. This decoupling between tracer signal and biological tumor load complicates intraoperative interpretation and limits the reliability of nanoparticle‐derived signals as direct surrogates for metastatic status. Addressing this limitation will require more detailed characterization of intranodal nanoparticle distribution at cellular and subnodal scales, including spatial relationships to tumor cells, stromal compartments, and immune niches. Future strategies may benefit from incorporating cell‐selective targeting ligands, tumor‐activated or enzyme‐responsive signal amplification mechanisms, or multiparametric readouts that integrate anatomical localization with functional or molecular cues. Such advances could enable a shift from purely localization‐driven mapping toward biologically informed SLN/TDLN assessment, enhancing the clinical interpretability and prognostic relevance of nanoparticle‐based lymphatic detection (Figure 6C).

The prolonged retention of magnetic or pigment‐based nanoparticles within SLNs supports their compatibility with delayed surgery, longitudinal staging, and multi‐step clinical workflows [56, 121]. However, because lymph nodes are dynamic immunological organs, whether and how persistent nanoparticle deposition affects nodal immune function remains an important unresolved question [119]. Most existing studies have focused on short‐term toxicity, procedural safety, or gross histopathological changes, whereas chronic immune activation, stromal remodeling, sustained macrophage engagement, and other functional changes within nanoparticle‐laden nodes have not been systematically examined. Since SLNs can serve as sites of immune priming and tumor–immune interaction, future work should determine whether prolonged nanoparticle residence could alter nodal physiology in ways that are not captured by conventional safety endpoints [122]. This question may become particularly relevant in contemporary treatment settings that incorporate neoadjuvant chemotherapy, immunotherapy, or repeated nodal interventions, where immune context and lymphatic integrity can influence therapeutic response. Future studies would therefore benefit from lymph node–centric safety frameworks that extend beyond acute tolerability to include long‐term immunophenotyping, cytokine profiling, and functional assessment of antigen presentation and lymphocyte trafficking. In parallel, biodegradable, metabolically cleavable, or clearance‐tunable nanoplatforms may help balance durable SLN/TDLN detectability with a lower potential immunological burden, thereby aligning nanoparticle persistence with both diagnostic utility and long‐term nodal homeostasis.

SLN/TDLN mapping is increasingly embedded within complex diagnostic and therapeutic trajectories, including neoadjuvant therapy, delayed surgery, and multimodal treatment integration. However, nanoparticle‐based detection strategies remain highly heterogeneous across tumor types, treatment sequences, and institutions, with substantial variability in tracer selection, injection timing, signal interpretation, and clinical decision thresholds [123, 124]. This lack of standardization limits reproducibility, complicates cross‐study comparison, and constrains the translation of nanotracer‐based SLN/TDLN detection from controlled investigations into routine oncologic practice. To enable broad clinical adoption, SLN/TDLN nanodetection must evolve from procedure‐specific implementations toward pathway‐aware and protocol‐driven paradigms. Leveraging the temporal stability, signal robustness, and multimodal compatibility of nanoparticle tracers, future studies should define context‐specific yet reproducible frameworks tailored to distinct clinical scenarios, such as pre‐neoadjuvant staging, post‐treatment restaging, or deferred sentinel‐node biopsy [125]. Establishing such standardized strategies will be critical for integrating nanoparticle‐enabled SLN/TDLN mapping as a structural component of oncologic workflows, rather than a technique‐dependent adjunct, thereby supporting more consistent staging, reduced surgical morbidity, and evidence‐based personalization of lymphatic management (Figure 6D).

## 9 Conclusion

Nanoparticle‐enabled SLN/TDLN detection is evolving from an adjunct to conventional dyes or radiotracers into a framework that reshapes lymph node assessment at both mechanistic and procedural levels. Through rational control of particle size, surface chemistry and physical signal properties, these nanosystems achieve regulated lymphatic transport, stable intranodal retention and imaging readouts with improved penetration and quantitative potential. Across material classes, convergent evidence indicates that nanoparticle tracers more faithfully reconstruct tumor‐draining nodal topology and substantially enhance the detection of occult metastatic involvement, particularly in anatomically complex regions or low‐burden disease.

Crucially, the value of nanoparticle‐based tracers extends beyond improvements in detection accuracy to their exceptional compatibility with real‐world clinical workflows. Enabled by persistent intranodal retention and durable magnetic or optical signatures, SLN/TDLN marking is no longer constrained by the traditional “same‐day injection–immediate surgery” paradigm, but can be flexibly integrated into neoadjuvant therapy, delayed operations and multi‐step diagnostic pathways. This temporal decoupling repositions lymphatic mapping as a pre‐planned diagnostic resource rather than a perioperative constraint. As nanoparticle tracers continue to mature in terms of manufacturability and clinical integration, their capacity to reduce unnecessary lymph node dissection and to support individualized staging decisions is likely to become increasingly central to precision oncologic management.

## Author Contributions

**Li Cui**: Conceptualization, Writing – Original Draft, Writing – Review & Editing, Supervision, Funding acquisition, Project administration, Methodology. **Xinyuan Zhao**: Conceptualization, Writing – Original Draft, Writing – Review & Editing, Supervision, Funding acquisition, Project administration, Methodology. **Xu Chen**: Writing – Original Draft, Visualization, Writing – Review & Editing. **Nina Li**: Writing – Original Draft, Visualization, Writing – Review & Editing. **Meiyan Zou**: Writing – Original Draft, Writing – Review & Editing, Visualization. **Zihao Zhou**: Writing – Original Draft, Writing – Review & Editing, Visualization. **Rongwei Xu**: Writing – Original Draft, Writing – Review & Editing, Visualization. **Weiyao Feng**: Writing – Original Draft, Writing – Review & Editing, Visualization.

## Funding

This work was supported by National Natural Science Foundation of China (82372905, 82573074), Guangdong Provincial Science and Technology Project Foundation (2022A0505050038), Young Top‐notch Talent of Pearl River Talent Plan (0920220228), Science and Technology Program of Guangzhou (No.2025A04J3464).

## Conflicts of Interest

The authors declare no conflicts of interest.

## Data Availability

Data sharing not applicable to this article as no datasets were generated or analysed during the current study.
